# Continued Menstruation during Pregnancy

**Published:** 1877-12-20

**Authors:** O. E. Newton

**Affiliations:** Cincinnati, O.


### CONTINUED MENSTRUATION DURING PREGNANCY.

BY O. E. NEWTON, M. D., CINCINNATI, O.

This condition can and does occur, but in very rare instances—so rare that one may practice for many years without having a case. In my practice I can recall to mind only two cases.

Attention will first be drawn to the fact that your patient is increasing in size, and apprehensive that she might be the subject of dropsy, it being supposed that so long as a woman menstruates, she cannot be pregnant.

If such a case should be presented to you, let the patient lie prone upon a bed or couch, with her person protected. Put your ear to the wall of the abdomen, and tap with your fingers on the opposite side. If you hear gurgling, or the noise of water, and the anterior wall can be easily pressed back, and it being also soft, you have probably all the evidence that is necessary to satisfy you. But if you find no symptoms of this kind, and find the walls firm, the form full and symmetrical, and resistance be present, you will be justified in the conclusion that, notwithstanding she menstruates regularly, she is pregnant. To be more sure, if agreeable to the patient, allow her to stand up, placing one foot upon an elevation to raise it higher than the other; pass your right finger in the vagina until it reaches the os tincæ; press the womb as high as you can, and allow it to again recede upon the finger. By this means you may determine if it be larger and heavier than an unimpregnated womb would be. You cannot, with the other usual symptoms of discrimination furnished, make any mistake as to the condition and indications. Such other symptoms are: Increase in size of the mammary glands; increased darkness of the areola; nausea in the morning; longing for, or antipathy to, any kind of food; change of temper, etc.—all of these symptoms are probably in cases of pregnancy, though all are not invariably found. But the absence of water, and the increased size and weight of the womb are too plain, usually, to allow you to make any mistake. Having made up your mind as near as you can, it is well still to protect yourself by saying: Pregnancy is almost certain, but it might be otherwise; and your duty is to be careful with your final opinion until the period of quickening has taken place, four to four and a half months after conception. If the patient does prove pregnant, your duty is to guard against all means calculated to produce miscarriage or premature labor, by general hygienic means as regards diet, exercise and the general strength of the patient, etc. This is all that is required.

You cannot prevent it, as it is a natural peculiarity of the patient. So inform her friends, but by all means make them understand that she is not the subject of dropsy, and give them the full reasons for knowing it; for if you do not fully and intelligibly convince them, they will surely leave you and seek other medical advice.

